# Post-COVID-19 encephalomyelitis

**DOI:** 10.1186/s42466-021-00113-4

**Published:** 2021-03-15

**Authors:** Ji-Won Kim, Nuran Abdullayev, Janina Neuneier, Gereon R. Fink, Helmar C. Lehmann

**Affiliations:** 1 Department of Neurology, University Hospital Cologne, Kerpener Straße 62, 50937 Cologne, Germany; 2 Department of Radiology, University Hospital Cologne, Cologne, Germany; 3 Cognitive Neuroscience, Institute of Neuroscience and Medicine (INM-3), Research Center Jülich, Jülich, Germany

**Keywords:** Myelitis, Neuromyelitis optica, Postinfectious, Autoimmunity

## Abstract

Since the outbreak of coronavirus disease 2019 (COVID-19), a growing number of cases of acute transverse myelitis associated with COVID-19 have been reported. Here, we present the case of a patient who developed sensory ataxia after COVID-19 with MR lesions suggestive for longitudinal myelitis and in the splenium of the corpus callosum. The patient was successfully treated with immunoadsorption.

In June 2020, a 46-year-old male patient was admitted to our hospital with sensory disturbances in both legs. Symptoms had started 2 weeks before with symmetrical prickling and numbness in his feet that gradually progressed to the buttocks. At admission, the patient reported feeling the same sensations in his fingers. The patient had been diagnosed with coronavirus disease 2019 (COVID-19) in the second half of March 2020, with flu-like symptoms persisting until early April. He had remained in quarantine until April 12 without any specific treatment and, since then, did not show any symptoms suggesting an infection. Neurological examination revealed a symmetrically reduced sense of vibration, fine touch, and proprioception in both legs, as well as an ataxic gait. Deep tendon reflexes were brisk. Blood testing did not show any sign of an acute infection, with normal levels of C-reactive protein (0.8 mg/l) and white blood cell count (5.28 × 10^9^/L). Magnetic resonance imaging (MRI) of the brain identified a small circular lesion in the corpus callosum’s splenium (Fig. 1b-c). MRI of the spinal cord revealed cervical canal stenosis at C4-C6 but no sign of compressive myelopathy or myelitis (Fig. 1d). Cerebrospinal fluid (CSF) analysis unveiled a mildly elevated protein level (0.94 g/l) and marginal mononuclear pleocytosis (6 leucocytes/μl), compatible with cervical canal stenosis. Though CSF testing standards for COVID-19 remain to be established, a limited number of cases have been reported where CSF SARS-CoV-2 antibodies were detected [1, 2]. Our patient was tested negative for CSF SARS-CoV-2 antibodies. Somatosensory evoked potentials (SEP) showed prolonged responses in the tibial nerve bilaterally. Since vitamin B12 levels were normal, the patient was treated with high-dose intravenous (IV) methylprednisolone (1250 mg) for 5 days. While repeated SEPs showed slightly more prolonged responses in the tibial nerves, the patient himself reported a gradual improvement of the sensory disturbances and was discharged.

**Fig. 1:**
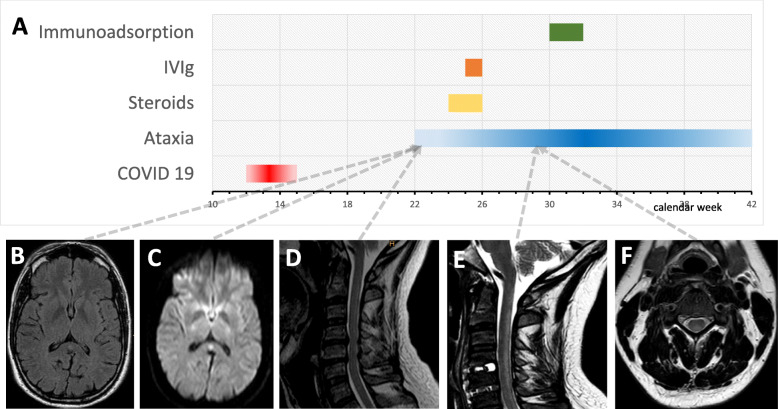
**a** Disease course and treatment of the patient. **b** Axial fluid-attenuated inversion recovery (FLAIR) and **c** axial diffusion-weighted imaging (DWI) show a hyperintensity in the splenium of the corpus callosum. **d** Sagittal T2 sequence of the cervical spine shows disc protrusion with spinal canal stenosis at C4-C6. **e** Postoperative sagittal and **f** axial T2 images of the cervical spine show a new hyperintensity in the dorsal columns extending from the cranial base to C6/7

One week later, the patient was re-admitted to our hospital due to worsening of the symptoms. Symptoms had progressed to a sensory tetra-syndrome with ataxic gait and truncal ataxia. Spinal MRI showed no changes. The patient was again treated with high-dose IV methylprednisolone for 3 days, followed by IV immunoglobulin for 5 days. As symptoms worsened nonetheless, we discussed that the cervical spinal canal stenosis at least partly contributed to them. After consultation of the department of orthopedic surgery, the patient who now used a wheelchair opted for a microsurgical discectomy at C4/5 and C5/6 with decompression of the spinal canal and osteophyte resection, despite being informed about the unclear clinical relevance of the cervical spinal canal stenosis. The surgery was performed on July 2 and the patient transferred to a neurological rehabilitation center.

At a first postoperative follow-up on July 22, the patient reported no changes in his symptoms before and after surgery with persisting sensory disturbances in all limbs and ataxia. Spinal MRI now revealed an extensively increased T2-signal in the dorsal columns, extending from the cranial base to C6/7 (Fig. 1e, f). CSF again revealed minimal pleocytosis (7 leucocytes/μl) but no oligoclonal bands. Vitamin B12 levels were repeatedly normal. Antibodies for myelin oligodendrocyte glycoprotein (MOG) and Aquaporin-4 (AQP4) were negative, too. The patient was treated with immunoadsorption for 6 days and again transferred to a neurological rehabilitation center. At the three-month follow-up, he reported a significant improvement during the ongoing rehabilitation.

Before July 23, repeated MRI scans of the spinal cord had not detected any sign of myelitis, even though the patient’s symptoms had persisted for almost 8 weeks. Initially, only a small lesion in the corpus callosum’s splenium had been detected. This finding resembles a previously published case report of mild encephalitis/encephalopathy with a reversible splenial lesion (MERS) associated with COVID-19, in which the patient presented mild ataxia [3]. To our knowledge, no case has been described with the combined occurrence of MERS and myelitis in association with COVID-19.

We considered whether the MRI lesion in the dorsal columns of the spinal cord could be a postoperative phenomenon. While this is a possibility, it was ruled unlikely as our patient displayed symptoms related to a lesion in the dorsal columns from the early stages of symptom onset prior to surgery. The length of the myelitis was suggestive of longitudinally extensive transverse myelitis (LETM) but did not feature the central cord predominance typical for LETM. Out of the core clinical characteristics for neuromyelitis optica spectrum disorders (NMOSD) our patient only showed acute myelitis and thus did not fulfill the diagnostic criteria for NMOSD without AQP4 antibodies [6]. Multiple sclerosis was considered unlikely due to the atypical MRI [5] and the absence of oligoclonal bands. Acute disseminated encephalomyelitis (ADEM) was assessed as unlikely because  behavioral changes, alterations in consciousness and large, multifocal brain lesions were absent in our patient [4].

In summary, our case report suggests that clinicians should consider MERS and myelitis when encountering a patient with ataxia during or after COVID-19. Treatment options may include IV immunoglobulin or immunoadsorption.

## Data Availability

The data generated and analyzed in this case report are available from the corresponding author on reasonable request.
